# Differential Neuroendocrine Responses and Dysregulation of the Hypothalamic–Pituitary–Adrenal Axis Following Repeated Mild Concussive Impacts and Blast Exposures in a Rat Model

**DOI:** 10.3390/brainsci15080847

**Published:** 2025-08-08

**Authors:** Rex Jeya Rajkumar Samdavid Thanapaul, Jishnu K. S. Krishnan, Manoj Y. Govindarajulu, Chetan Y. Pundkar, Gaurav Phuyal, Joseph B. Long, Peethambaran Arun

**Affiliations:** Blast-Induced Neurotrauma Branch, Center for Military Psychiatry and Neuroscience, Walter Reed Army Institute of Research, Silver Spring, MD 20910, USA; rexjeyarajkumar.samdavidthanapaul.ctr@health.mil (R.J.R.S.T.); jkkrishnan@alaska.edu (J.K.S.K.); manoj.y.govindarajulu.ctr@health.mil (M.Y.G.); chetan.y.pundkar.ctr@health.mil (C.Y.P.); gaurav.phuyal.ctr@health.mil (G.P.); josephlong815@gmail.com (J.B.L.)

**Keywords:** traumatic brain injury, blast exposure, mild concussive impact, HPA axis, neuroendocrine dysfunction

## Abstract

Traumatic brain injury (TBI) remains a significant public health concern, particularly among military personnel and contact sport athletes who are frequently exposed to repeated blast overpressure waves and mild concussive impacts, respectively. While moderate and severe TBIs have been extensively studied, the long-term neuroendocrine consequences of mild, repetitive brain trauma are poorly understood. In this study, we investigated the temporal dynamics of hypothalamic–pituitary–adrenal (HPA) axis dysregulation following repeated mild concussive head impacts and blast exposures using two clinically relevant rodent models. Male Sprague-Dawley rats were subjected to repeated mild concussive impacts using a modified weight drop model or repeated blast exposures using an advanced blast simulator. Plasma levels of adrenocorticotropic hormone (ACTH) and corticosterone were measured on days 1 and 30 post-injuries. Our findings revealed that repeated blast exposures induced elevation of plasma ACTH and corticosterone on days 1 and 30 post-blasts. After the repeated mild concussive impacts, increased plasma levels of corticosterone were observed on days 1 and 30, but ACTH levels were increased only on day 30. This study is among the first to directly compare neuroendocrine outcomes of repeated mild concussive impacts and blast exposures within a unified experimental framework. Our findings demonstrate distinct temporal trajectories of HPA axis dysregulation depending on injury type and highlight plasma levels of ACTH and corticosterone as potential biomarkers of subclinical brain trauma. These insights may inform early diagnostic approaches and therapeutic strategies aimed at mitigating long-term stress-related complications following mild traumatic brain injuries.

## 1. Introduction

Traumatic brain injury (TBI) is a primary global public health concern and a leading cause of long-term neurological disability in both military and civilian populations [1,2]. While the effects of moderate and severe TBI have been extensively characterized, the enduring neuroendocrine consequences of mild, repetitive brain trauma, particularly mild concussive impacts and blast exposures, remain insufficiently understood [3]. This gap is especially critical given the high prevalence of such injuries in contact sports and/or modern military conflicts [4,5].

Mild concussive head impacts, defined as head traumas that do not result in overt clinical symptoms or loss of consciousness, are particularly concerning due to their cumulative nature in contact sports [6]. These impacts often go unrecognized and untreated, yet accumulating evidence links them to late-onset neurodegenerative conditions such as chronic traumatic encephalopathy (CTE) [7]. In parallel, blast-induced traumatic brain injury (bTBI), a hallmark injury of recent military engagements, has been shown to cause widespread physiological and neurological alterations, including persistent disruption of the hypothalamic–pituitary–adrenal (HPA) axis [8,9].

The HPA axis plays a central role in coordinating the body’s physiological response to stress, and its dysregulation has been increasingly implicated in the pathogenesis of various post-traumatic neuropsychiatric and cognitive disorders [10]. Both mild concussive trauma and blast exposure are capable of altering the secretion of key stress-responsive hormones, such as adrenocorticotropic hormone (ACTH) and corticosterone [11,12]. While reduced ACTH levels suggestive of hypopituitarism have been observed in non-blast-related TBI, elevated ACTH levels have been reported in acute ischemic stroke and in specific brain trauma contexts, underscoring the complexity and context-specific nature of endocrine dysregulation following neural injury [3,13].

Despite these findings, a significant gap remains in our understanding of the temporal trajectory of neuroendocrine responses following repeated mild concussive impacts and blast exposures, particularly in the absence of overt neurological symptoms. Notably, few, if any, studies have conducted direct, side-by-side comparisons of these two injury types using standardized experimental conditions [14,15].

To address this critical gap, the present study utilizes two well-established and clinically relevant rodent models: a modified weight-drop model to simulate repeated mild concussive head impacts, and primary bTBI using an advanced blast simulator (ABS) to model repeated blast exposures [16]. These models emulate real-world scenarios commonly encountered by athletes and/or military personnel, where repeated mild trauma often accumulates without immediate clinical recognition. By measuring plasma ACTH and corticosterone levels at multiple time points (days 1 and 30 post-injury), this study aims to delineate both acute and sub-acute neuroendocrine outcomes.

The primary objective is to investigate and compare the temporal dynamics of HPA axis disruption following repeated mild concussive impacts and blast exposures. Specifically, this study seeks to (1) characterize changes in ACTH and corticosterone levels after repeated mild concussive impacts; (2) evaluate hormonal alterations following repeated blast exposures; and (3) identify shared and distinct neuroendocrine response patterns between these injury types.

## 2. Materials and Methods

All animal procedures in this study were conducted in strict accordance with the Animal Welfare Act and applicable federal regulations, following the *Guide for the Care and Use of Laboratory Animals*. All protocols were reviewed and approved by the Institutional Animal Care and Use Committee (IACUC) at the Walter Reed Army Institute of Research (WRAIR), ensuring ethical and humane treatment of animals. All experiments were conducted in an AAALAC-accredited facility under controlled environmental conditions (20–22 °C, 12:12 h light/dark cycle), with animals housed in individually ventilated cages and provided ad libitum access to chlorinated water and standard rodent chow (Prolab IsoPro RMH3000, LabDiet, St. Louis, MO, USA).

Male Sprague-Dawley rats (Charles River Laboratories, Wilmington, MA, USA), aged 9–10 weeks and weighing 300–350 g, were used for all experimental groups. Animals were randomly assigned to one of three groups: sham, repeated weight drop (WD), or repeated blast (RB). To model mild concussive head trauma, a modified Marmarou weight-drop model was used [17]. A 450 g cylindrical weight was dropped from a height of 75 cm onto a metal disc affixed to the isoflurane-anesthetized rat’s cranium to generate mild concussive impacts as described [18]. For repeated mild concussive events, the WD procedure was repeated after one week. Sham animals had only surgical implantation of the disc and were not exposed to blasts or subject to WD. A group of rats assigned for blast injury also had the surgical implantation of the metal disc and was exposed to a tightly coupled repeated blast (19 psi with a positive phase duration of 2 min) using the ABS as described earlier on the eighth day of the first surgical procedure [16,18].

Terminal blood collection was performed via cardiac puncture under deep anesthesia (5% isoflurane) on either day 1 or day 30 post-injury. To minimize circadian variability in hormone levels, all samples were collected between 08:00 and 10:00 a.m. during the early light phase of the 12:12 h light/dark cycle. Plasma was isolated and stored at −80 °C until further analysis. Plasma concentrations of adrenocorticotropic hormone (ACTH) and corticosterone were measured using commercially available enzyme-linked immunosorbent assay (ELISA) kits from Novus Biological (Centennial, CO, USA) and Abcam (Waltham, MA, USA), respectively, following the manufacturers’ protocols. All samples were assayed in triplicate, and absorbance was measured using a microplate reader (BioTek Instruments, Winooski, VT, USA). Hormone concentrations were determined based on standard curves generated from each assay.

Each experimental group included 9–12 animals per time point. Statistical analyses were performed using GraphPad Prism version 9.5.1 (GraphPad Software, San Diego, CA, USA). Data were analyzed by two-way analysis of variance (ANOVA) followed by Tukey’s post hoc test for multiple comparisons. Results are expressed as mean ± standard error of the mean (SEM). Statistical significance was defined as *p* < 0.05, and *p* < 0.01 was considered highly significant.

## 3. Results

### 3.1. ACTH Levels Following Mild Concussive Impacts and Blast Exposures

To evaluate hypothalamic–pituitary–adrenal (HPA) axis activity, plasma ACTH concentrations were measured on days 1 and 30 post-injury across three treatment groups: sham (blue), repeated mild concussive impacts using weight drop (WD), and repeated blasts using the ABS (RB).

**Figure 1:**
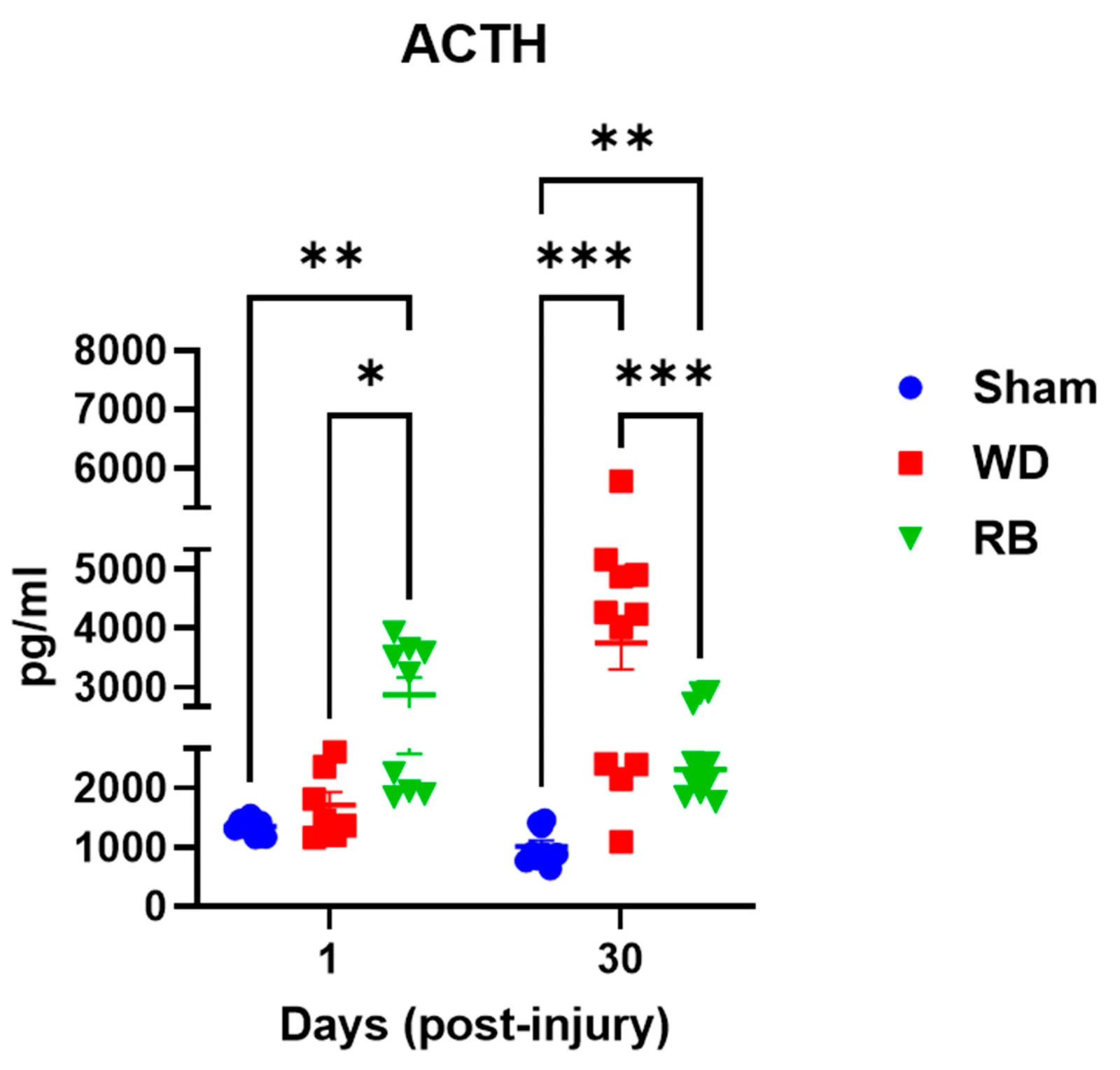
Plasma ACTH levels following repeated mild concussive impacts and blast exposures. Plasma ACTH concentrations were quantified on days 1 and 30 post-injury in rats exposed to repeated weight drop (WD, red), repeated blast (RB, green), or sham procedures (blue). Data are presented as mean ± SEM (*n* = 9–12 per group). Statistical analysis was performed using two-way ANOVA followed by Tukey’s post hoc test. * *p* < 0.05, ** *p* < 0.01, *** *p* < 0.001.

On day 1, ACTH levels were significantly elevated in the RB group compared to both the sham (*p* < 0.01) and WD groups (*p* < 0.05) (Figure 1). The WD group also exhibited a moderate but statistically significant increase compared to sham (*p* < 0.05), indicating a differential early neuroendocrine response. These results suggest that repeated blast exposure provokes a robust acute HPA axis activation, while repeated mild concussive impacts result in a comparatively lower but measurable early elevation.

By day 30, both RB and WD groups maintained significantly elevated ACTH levels relative to the sham group (RB: *p* < 0.01; WD: *p* < 0.001). Notably, the WD group exhibited the highest ACTH concentration overall, significantly exceeding levels in both sham and RB groups (*p* < 0.001). This indicates a delayed but sustained neuroendocrine activation following repeated concussive impacts. The RB group also demonstrated prolonged HPA axis engagement, with ACTH remaining significantly elevated compared to sham (*p* < 0.01).

Collectively, these findings reveal distinct temporal profiles of ACTH regulation that depend on the type of injury. Repeated blast exposure triggers an immediate neuroendocrine surge, while repeated mild concussive trauma leads to a delayed and more prolonged HPA axis response.

### 3.2. Corticosterone Levels Following Mild Concussive Impacts and Blast Exposures

Plasma corticosterone concentrations were measured on days 1 and 30 post-injury as an index of HPA axis activation across sham, repeated weight drop (WD), and repeated blast (RB) groups.

**Figure 2:**
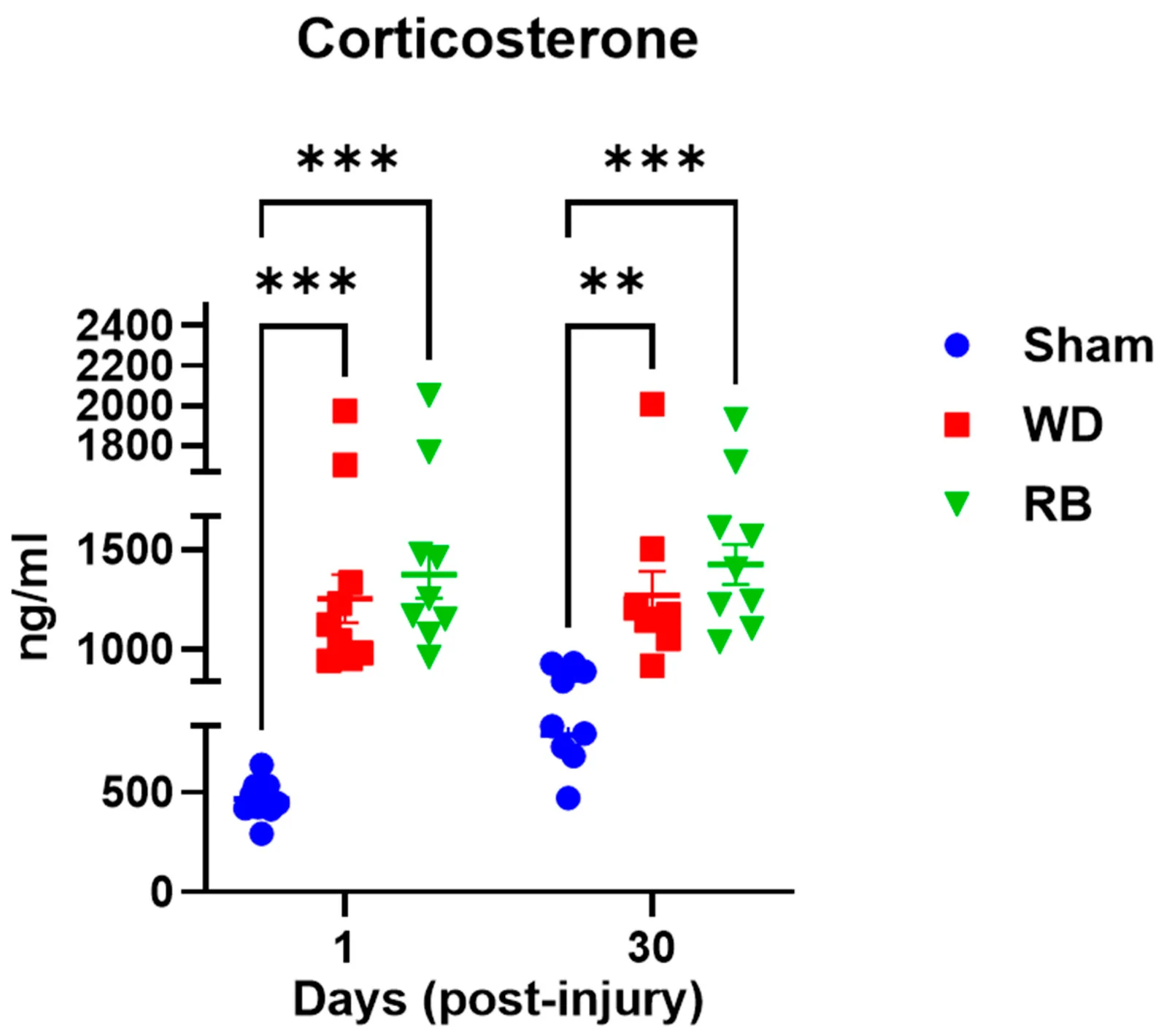
Plasma corticosterone levels following repeated mild concussive impacts and blast exposures. Corticosterone concentrations were measured on days 1 and 30 post-injury in rats subjected to repeated weight drop (WD, red) or repeated blast (RB, green), compared to sham controls (blue). Data are expressed as mean ± SEM (n = 9–12 per group). Statistical analysis was performed using two-way ANOVA followed by Tukey’s post hoc test. * *p* < 0.05, ** *p* < 0.01, *** *p* < 0.001.

On day 1, corticosterone levels were significantly elevated in both the WD and RB groups compared to sham controls (*p* < 0.001 for both) (Figure 2). No significant difference was detected between the WD and RB groups (*p* > 0.05), indicating that repeated mild concussive impacts and repeated blast exposures both elicit a robust and comparable acute stress hormone response.

By day 30, corticosterone levels remained significantly elevated in the WD (*p* < 0.01) and RB (*p* < 0.001) groups relative to sham. Again, no significant difference was observed between the WD and RB groups (*p* > 0.05), suggesting that both injury modalities induce sustained HPA axis dysregulation extending into the sub-acute phase.

Collectively, these results demonstrate that repeated concussive impacts and blast exposures produce long-lasting activation of the HPA axis, as evidenced by persistent elevation of corticosterone levels. These findings underscore the chronic neuroendocrine impact of repetitive injuries, regardless of the mechanism of injury.

## 4. Discussion

Traumatic brain injuries (TBIs), especially those caused by repeated mild concussive impacts and blast exposures, pose a growing threat to neurological and endocrine health, particularly among military personnel and/or athletes in contact sports [19,20,21]. While moderate to severe TBIs have been extensively researched, the long-term neuroendocrine effects of mild and repetitive brain trauma are still not well understood. This study aims to fill that important gap by directly comparing HPA axis dysregulation following repeated mild concussive impacts and blast exposures through two well-validated rodent models. Our results demonstrate distinct temporal patterns in ACTH and corticosterone secretion that differ by injury type, suggesting that varying mechanisms may contribute to neuroendocrine dysfunction after mild traumatic exposures.

A key finding of this study is that repeated blast exposure triggered a robust acute increase in both ACTH and corticosterone levels on day 1. In contrast, repeated mild concussive impacts resulted in a delayed but sustained elevation in ACTH with significantly high levels on day 30. These observations align with emerging human and animal studies. For instance, altered cortisol rhythms have been reported in military veterans with a history of blast exposure [22,23,24], and blast injuries in rodents have been shown to produce chronic stress responses and behavioral dysregulation [25,26,27]. Our findings extend this understanding by demonstrating that mild concussive trauma, when repeated, can elicit comparable or even greater neuroendocrine disruption than blast exposure, especially at later time points.

The temporal disruption in ACTH responses between types of injuries carries important mechanistic implications. The acute elevations of ACTH and corticosterone observed after repeated blast exposure may reflect the direct and immediate activation or disinhibition of the entire HPA axis, potentially involving diffuse neural injury, pituitary dysfunction, or systemic inflammation [28,29]. Conversely, the delayed ACTH elevation in the weight drop model, despite an acute rise in corticosterone, suggests that early adrenal activation may occur independently of pituitary drive, possibly due to increased adrenal sensitivity or peripheral stress signaling [30,31,32]. The later rise in ACTH could indicate pituitary hyperactivity or impaired glucocorticoid feedback, potentially resulting from cumulative hypothalamic damage or receptor desensitization [33]. These patterns suggest that the specific mechanical force, whether blast wave or blunt impact, may differentially affect components of the HPA axis or trigger distinct secondary injury cascades, such as neuroinflammation [34].

This study also offers translationally relevant insights by modeling two distinct, yet common types of traumas under matched experimental conditions. Unlike previous studies that examine either blast or impact in isolation, our approach allows for a direct comparison of endocrine outcomes. Both repeated injury models resulted in sustained elevations of corticosterone. However, the repeated mild concussive impacts resulted in a sub-acute increase in ACTH compared to the acute changes, suggesting that mild concussive impacts may have a more pronounced effect on pituitary function at later time points post-injury. This divergence could be attributed to differences in the propagation of mechanical force, with blast injuries inducing acute diffuse axonal and vascular damage. At the same time, mild concussive impacts are more likely to affect superficial cortical or subcortical structures initially, leading to gradual diffuse axonal injuries in structures such as the hypothalamus [19,20,21,22,23,24,25,26,27,28,29,30,31,32,33,34,35].

Our results position ACTH and corticosterone as promising, yet imperfect, biomarkers for detecting subclinical brain trauma. This is particularly important because mild and repetitive TBIs often present without overt clinical symptoms, leading to “invisible” disabilities such as fatigue, mood disturbances, and cognitive decline [36,37]. Identifying physiological markers that precede these symptoms could facilitate early diagnosis and intervention. However, the clinical utility of ACTH and corticosterone is complicated by their pulsatile secretion, diurnal variation, assay limitations, and overlap with comorbid conditions like post-traumatic stress disorder (PTSD) [38,39,40]. Single hormone measurements may not accurately reflect systemic glucocorticoid status, so standardized testing protocols, including early morning sampling and stimulation tests, are necessary for clinical application.

Importantly, while we used standardized, validated models to simulate mild brain trauma, a key limitation of the current study is the absence of direct calibration of injury severity between the blast and weight drop paradigms. Although both models are designed to produce clinically relevant mild TBIs without apparent neurological deficits, differences in HPA axis outcomes may partly reflect variations in injury severity rather than solely biomechanical mechanisms. In prior work, using these same injury protocols [18], we observed similar behavioral and cognitive impairments across the two models, supporting the idea that the overall injury burden may be comparable. However, further studies with quantitative neuropathological and neurobehavioral assessments are needed to definitively determine severity equivalence and isolate the biomechanical factors contributing to HPA axis dysregulation.

Additional limitations warrant consideration. First, the exclusive use of male rats limits the generalizability of our findings, as sex differences in HPA axis function and TBI outcomes are well-established. Including female subjects in future studies will be essential to identify sex-specific vulnerabilities. Second, although we observed significant hormonal changes, behavioral assessments such as cognitive and affective testing were not conducted. These would provide critical functional context for interpreting neuroendocrine alterations. Third, our study focused on peripheral hormone levels and did not include functional assays, such as dexamethasone suppression or CRH/ACTH stimulation tests, which are feasible in rats and would offer direct insight into feedback sensitivity and central HPA axis responsiveness. Fourth, molecular analyses of central regulatory structures such as the hypothalamus, and pituitary and adrenal glands were not performed, precluding mechanistic understanding of upstream dysregulation. Gene and protein expression profiling in these regions will be necessary in future work to link peripheral endocrine changes with central neurobiological alterations. Finally, while ACTH and corticosterone serve as indicators of systemic stress, their diagnostic specificity for mild TBI remains limited, particularly in the presence of comorbidities or overlapping stress-related conditions.

Looking ahead, future studies should broaden the biological scope by including both sexes and evaluating longer post-injury windows, such as 60 or 90 days, to determine the persistence and recovery trajectory of endocrine dysfunction. Incorporating intermediate time points will enhance the resolution of the hormonal response over time. Behavioral assessments, such as tests for memory, anxiety, and locomotion, should be integrated to directly connect endocrine disruption with functional deficits. In parallel, molecular and histological studies of the HPA axis organs are necessary to identify underlying mechanisms, including neuronal injury, inflammation, or receptor alterations. Therapeutically, investigating interventions such as glucocorticoid receptor modulators or anti-inflammatory agents could help reverse or alleviate chronic HPA axis dysfunction. Broader biomarker panels that include growth hormones, thyroid hormones, gonadotropins, and neuroinflammatory markers should also be explored to develop a comprehensive diagnostic strategy.

In conclusion, this study reveals that both repeated mild concussive impacts and repeated blast exposures lead to significant and lasting dysregulation of the HPA axis. The distinct temporal profiles of ACTH and corticosterone highlight mechanistic differences between injury modalities and reinforce the importance of injury repetition in determining long-term outcomes. These findings have critical implications for early diagnosis, risk stratification, and therapeutic development. ACTH and corticosterone emerge as promising indicators of subclinical brain trauma, particularly in populations frequently exposed to repeated mild TBIs. Thus, this work lays a scientific foundation for future efforts aimed at developing personalized monitoring and intervention strategies to reduce the burden of chronic stress-related comorbidities such as CTE and PTSD in vulnerable individuals.

## Data Availability

The original contributions presented in this study are included in the article. Further inquiries can be directed to the corresponding author.

## Disclaimer

Material has been reviewed by the Walter Reed Army Institute of Research. There is no objection to its presentation and/or publication. The opinions or assertions contained herein are the private views of the author and are not to be construed as official, or as reflecting true views of the Department of the Army or the Department of Defense. Research was conducted under an IACUC-approved animal use protocol in an AAALAC International-accredited facility with Public Health Services Animal Welfare Assurance and in compliance with the Animal Welfare Act and other federal statutes and regulations relating to laboratory animals.

## Abbreviations

The following abbreviations are used in this manuscript:

TBI	Traumatic Brain Injury
bTBI	Blast-Induced Traumatic Brain Injury
HPA Axis	Hypothalamic–Pituitary–Adrenal Axis
ACTH	Adrenocorticotropic Hormone
WD	Weight Drop
RB	Repeated Blast
ABS	Advanced Blast Simulator
CTE	Chronic Traumatic Encephalopathy
PTSD	Post-Traumatic Stress Disorder
ELISA	Enzyme-Linked Immunosorbent Assay
IACUC	Institutional Animal Care and Use Committee
AAALAC	Association for Assessment and Accreditation of Laboratory Animal Care
SEM	Standard Error of the Mean
ANOVA	Analysis of Variance
CRH	Corticotropin-Releasing Hormone
